# Self-Reported Cognitive Function in Persons with Nonneurological Chronic Diseases: A Systematic Review

**DOI:** 10.1155/2022/5803337

**Published:** 2022-03-31

**Authors:** Heather Cuevas, Valerie Danesh, Ashley Henneghan

**Affiliations:** ^1^School of Nursing, University of Texas at Austin, 1710 Red River St. Mail Code D0100, Austin 78712, TX, USA; ^2^Center for Applied Health Research, Baylor Scott & White Research Institute, 2401 S. 31st St Temple, Dallas 76508, TX, USA; ^3^Dell Medical School, Department of Oncology, University of Texas at Austin, Austin, TX, USA

## Abstract

**Background:**

Globally, one in three adults has a chronic condition. Many chronic diseases that are not neurological in nature (e.g., diabetes and heart failure) are increasingly associated with cognitive symptoms. However, the instruments used to assess cognitive symptoms in those with nonneurologic chronic illness are heterogeneous, and questions remain as to how cognitive symptoms may be related to demographic and clinical outcome variables, neurocognitive test performance, and other patient-reported outcomes. In this review, we describe associations among self-reported cognitive function, cognitive performance, and additional patient-reported outcomes as well as how cognitive symptoms are measured in nonneurologic chronic illness.

**Method:**

Multiple databases (PubMed, Medline, CINAHL, PsycInfo, EMBASE, SCOPUS, the Cochrane Library, and Academic Search Complete) were searched for studies from 1990 to 2020 that provided data on self-reported cognitive symptoms in those with nonneurological chronic conditions. Initial search yielded 304 articles, of which 32 met inclusion criteria. Quality assessment was conducted using the Critical Appraisal Skills Programme.

**Results:**

Thirty-two total studies were included: twenty cross-sectional, 10 longitudinal, and 2 randomized controlled trials. The tools used to assess self-reported cognitive function in the studies were heterogeneous: 28 unique tools were used. Thirty studies examined associations among self-reported cognitive function and other patient-reported outcomes. In 19 there were significant associations. Six studies showed no significant associations between neuropsychological tests and self-reported cognitive function; another 6 studies found a significant association.

**Conclusion:**

Tools to assess cognitive symptoms were heterogeneous. In most studies, self-reported cognitive symptoms were not correlated with neuropsychological test results, but the majority of studies found a strong association between self-reported cognitive function and other patient-reported outcomes. *Implications*. Consensus on measuring cognitive symptoms would facilitate cross-study comparisons and facilitate scientific progress in those with nonneurological chronic conditions. Based on these results, there is a need to establish a standardized approach for self-reported cognitive function measurement in patients with nonneurologic chronic illness.

## 1. Introduction

National surveys suggest that more than 26% of older adults are concerned about a potential diagnosis of Alzheimer's, and more than 50% are concerned about becoming a burden on family because of future cognitive problems [1, 2]. Some cognitive decline is expected in older adults, but cognitive changes that impair one's ability to function in middle to late adulthood are unexpected. These changes are complex and multifaceted, especially in those with nonneurologic chronic conditions with known cognitive risk factors (e.g., diabetes, cardiovascular disease, and cancer) [3, 4]. However, despite risk factors and the prevalence of cognitive changes in those with nonneurologic chronic conditions, less is known about cognitive function in such populations.

The effects and impact of cognitive dysfunction on day-to-day life such as difficulties in memory and deficits in attention are difficult to assess with standard neuropsychological tests [5]. Individuals' perspectives are therefore critical to our understanding of cognitive symptoms, not only because perceived cognitive decline may be a precursor to mild cognitive development and dementia [6], but also because self-reported cognitive function captures the impact of cognitive symptoms on daily function. At least 20% of people 45 years and older with one chronic disease report having cognitive problems, and this prevalence may be higher for those with specific conditions [7]. Those who have had a stroke, a history of heart disease, or chronic obstructive pulmonary disease have a higher occurrence of self-reported cognitive symptoms than do those without those diseases [8]. For example, 27.1% of adults aged 45–65 years who have coronary artery disease report subjective cognitive problems, whereas in healthy adults 65 and older, the prevalence is 18.7% [7]. The presence of midlife self-reported cognitive dysfunction can be a risk for dementia, sometimes presenting before objective impairments are found with neuropsychological tests [9]. In addition, self-reported cognitive dysfunction can impact daily self-management of chronic conditions such as diabetes [10, 11] as well as quality of life [12, 13]. As a result, research on self-reported cognitive dysfunction in persons at risk for mild cognitive impairment has increased [14].

In this review, we describe associations among self-reported cognitive function (SRCF), cognitive performance, and additional patient-reported outcomes as well as how cognitive symptoms are measured in nonneurologic chronic illness.

## 2. Methods

The Preferred Reporting Items for Systematic Reviews and Meta-Analyses (PRISMA) statement, including the PRISMA 27-item checklist of essential review characteristics, informed the procedures for this review [15] (Supplementary Material A). The review protocol was registered with PROSPERO (CRD4202146706) per guidance from the Cochrane Collaborative [15].

### 2.1. Data Sources, Search Strategy, and Selection

The following databases were searched for articles related to SRCF in peer-reviewed journals from January 1990 through October 2020: CINAHL, MedLine, PubMed, PsycInfo, EMBASE, SCOPUS, the Cochrane Library, and Academic Search Complete.

Broad search terms (MeSH) and synonyms were used including *subjective cognitive complaints*, *perceived cognitive problems*, *chronic conditions*, and *chronic disease* (Supplementary Material B). Citations of all relevant studies were also reviewed. MedLine was searched first, and resulting syntax and headings were used to search the other databases. Key inclusion criteria were as follows: (1) use of a self-report measure of cognitive function (e.g., perceived cognitive issues, symptoms of cognitive problems, memory complaints); (2) participants 18 years of age or older; and (3) participants diagnosed with one or more nonneurologic chronic conditions (e.g., type 2 diabetes, coronary artery disease, and obstructive pulmonary disease). All quantitative study designs—randomized controlled trials (RCTs), cross-sectional studies, and longitudinal studies—were included. Exclusion criteria were as follows: (1) study participants diagnosed with neurologic chronic conditions, such as dementia, stroke, HIV-associated cognitive disorders, and central nervous system disorders; (2) publications that were not peer-reviewed or not written in English; and (3) posters, review papers, letters, and conference proceedings. We also excluded studies of those with chemotherapy-related cognitive dysfunction, because comprehensive reviews have examined self-reported cognitive function following chemotherapy treatment [16, 17]. All titles, abstracts, and full texts of the studies were independently screened by two reviewers, and disagreements were settled to ensure the studies' eligibility.

### 2.2. Data Extraction

Data, extracted by all authors, included author, year of publication, research design, data collection time points, purpose of the study, study setting, sample characteristics, measures of SRCF and primary variables, and associations with objective neuropsychological tests. The first and second reviewers double-verified the extracted data for accuracy. When data needed for extraction were missing, the first author contacted the authors of the study via e-mail to request the data (Supplementary Material C).

### 2.3. Quality Appraisal

The Critical Appraisals Skills Programme (CASP) [18] was used to assess the quality of the included studies. In this review, randomized controlled trials (RCTs) were assessed using the CASP RCT checklist, and the remaining studies were evaluated using the CASP cohort study checklist.

### 2.4. Synthesis

Meta-analysis was not feasible, due to the variability among studies in design, measures of self-reported cognitive function, and outcome variables. The data were instead analyzed using Popay et al. [19] methods for narrative synthesis in systematic reviews.

## 3. Results

### 3.1. Search

**Figure 1:**
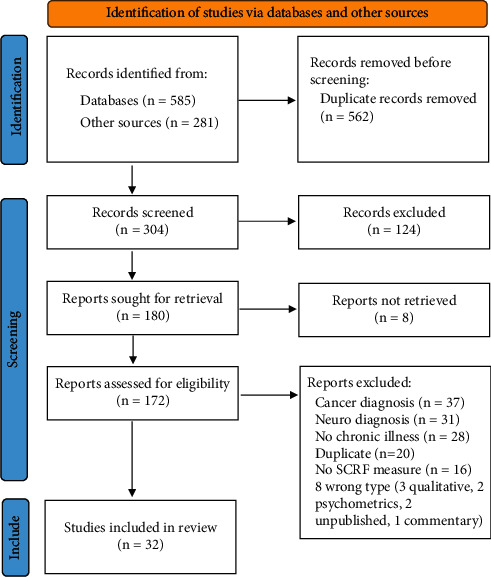
PRISMA flow diagram.

Eight hundred and sixty-six eligible studies were retrieved from the databases. After duplicates (*n* = 562) were removed, 304 titles remained and were imported to an online platform from Covidence (https://covidence.org) for independent screening and data extraction. A total of 32 studies were included in the final analysis (See Figure 1 PRISMA flow diagram for details of screening.).

### 3.2. Risk of Bias/Quality Assessment

Two studies were RCTs; for both studies, all 11 items on the CASP [18] RCT checklist indicated relatively high study quality. Of the remaining studies, a few had high scores, with no quality issues recognized (*n* = 8) or one or two low (“No”) quality scores (*n* = 4). Most of the “No” scores were related to confounding factors (*n* = 3) and bias minimization (*n* = 1) (Supplementary Material D).

### 3.3. Characteristics of Included Studies

Of the 32 studies included in the analysis [20–51], 20 (63%) were cross-sectional, 10 (31%) were longitudinal, and two (6%) were RCTs (Table 1). Of the two RCTs, one investigated the effects of a computerized cognitive training intervention in participants with chronic pain [23]. The other examined the effects of cognitive behavioral therapy on cognitive impairment, both objective and subjective [38]. Six studies compared participants with healthy controls [21, 25, 39, 42, 45, 49]. Four other studies used specific comparators: (1) treatment with steroids versus nontreatment [30]; (2) two or more chronic conditions versus one or none [35]; (3) amputation for vascular or nonvascular etiologies [41]; and (4) treatment with erythropoietin versus nontreatment [43]. Follow-up in the longitudinal studies ranged from 1 week to 2 years.

#### 3.3.1. Study Populations

Sample sizes ranged from 26 to 11,379 with a mean age range of 30 to 78.5 years. The most common chronic conditions included coronary artery disease (10 studies), HIV (4 studies), and chronic pain (4 studies). Fifteen studies had a majority of females in the sample, 15 were majority males, and 2 were evenly split. Seventeen studies did not report the ethnic makeup of the samples. Sixteen of the studies were conducted in the U.S., and four were conducted in The Netherlands (See Table 1 and Supplementary Material E).

#### 3.3.2. Self-Reported Cognitive Function Measures

Twenty-eight different tools were used to assess SRCF across the 32 studies. The most common were the Cognitive Failures Questionnaire (five studies) and the Cognitive Difficulties Scale (three studies). Other tools included the Everyday Memory Questionnaire, the Cognitive Complaints Inventory, the Behavior Rating Inventory of Executive Function, and the Health Complaints Scale. Four were sets of author-derived questions, with items such as “Do you have any complaints concerning your memory?” (See Supplementary Material F). Three studies did not report the cognitive domains assessed by the SCRF measure [43, 47, 49] and two used “global” SCRF tools [30, 42]. The remaining studies ranged from measuring one domain (e.g., only executive function) [23, 39] to seven domains [26]. Ten studies [21, 25, 28, 31, 32, 36, 37, 44, 48, 51] reported reliability of the SCRF tools with Cronbach's alphas ranging from .67 to .8 for the Health Complaints Scale–Concentration subscale to .96 for the Cognitive Difficulties Scale. Items on each tool ranged from three [39] to 95 [21].

#### 3.3.3. Reporting of Other Patient-Reported Outcomes

Thirty studies included assessment of other self-reported outcomes. The most common were the Beck Depression Inventory (6 studies) and the Hospital Anxiety and Depression Scale (3 studies). Thirteen studies (41%) included objective neuropsychological testing. The neuropsychological tests ranged from screening tests to computerized assessments to comprehensive batteries. Only one study included imaging [32].

### 3.4. Primary Study Results

Descriptions of study outcomes varied widely in design, SRCF related endpoints, and measures. Therefore, a meta-analysis could not be conducted. In some studies, only descriptive results of self-report measures were reported (e.g., means, percentages). Other studies gave more in-depth results including (1) comparison of symptoms of cognitive problems, usually between participants with a chronic illness and those who did not; (2) cognitive changes over time in longitudinal studies; and (3) differences in cognitive symptoms between groups in RCTs of interventions. Therefore, the number of studies was insufficient to conduct a meta-analysis.

#### 3.4.1. Demographics and Self-Reported Cognitive Function

Three studies [33, 37, 48] focused mainly on participants' demographic characteristics in relation to self-perceived cognitive function. One of these studies [37] investigated the relationship further, using longitudinal data collected at 12 months. Two of the three studies [33, 37] found a significant relationship between unemployment/early retirement/homemaker and increased cognitive complaints. Middle age (45–54 years) was also associated with more cognitive complaints [33], and older age (55 and older) was associated with more cognitive complaints [37, 48].

#### 3.4.2. Self-Reported Cognitive Function and Neuropsychological Tests

Nine studies (28%) compared objective neuropsychological tests of cognitive function with SRCF [22, 24, 25, 27, 29, 32, 34, 42, 46] with Pearson's *r* correlations ranging from −24 to −27 [25] to .44 to .85 [22]. Two of them found no significant relationships between self-report and objective measures [24, 27]. Four others [22, 25, 29, 32] identified relationships between specific cognitive domains and perceived function. In two of those four, memory concerns were significantly related to objective memory test performance [22, 32]. In another, greater overall perceived problems were associated with worse scores on executive function, processing speed, and language measurements [25]. The last found that higher scores on the Montreal Cognitive Assessment were significantly related to fewer everyday cognitive symptoms [29]. Steinbusch et al. [46] followed cognitive function over time and found that higher baseline cognitive complaints were significantly related to lower cognitive ability at 12 months. Jackson and Cooper [34] found that a diagnosis of hypertension was associated with worse objective cognitive function over time in those with SRCF than in those without. Similarly, Nguyen et al. [42] showed that community dwelling older adults who had hypertension and memory concerns had worse objective performance on cognitive tests than did nonhypertensive participants with memory complaints.

#### 3.4.3. Other Patient-Reported Outcomes and SRCF

A number of additional patient-reported outcomes were associated with SRCF in samples with various nonneurologic chronic conditions. Two studies reported findings from separate samples undergoing interventions for cardiovascular disease—percutaneous coronary interventions (PCIs) and coronary artery bypass surgery (CABG) [28, 31]. For those undergoing PCI, poorer perceived cognitive function was associated with poorer quality of life independently of demographics, fatigue, mood, and other clinical variables [28]. In the sample undergoing CABG, baseline cognitive complaints predicted a higher rate of negative emotional symptoms at 5 months [31]. For those with cardiovascular disease, but not undergoing any cardiac interventions, worse SRCF had a significant association with worse quality of life [36]. For patients with chronic pain, female gender, pain intensity, catastrophizing, posttraumatic stress disorder, depression, location of pain, and fatigue were positively associated with cognitive complaints. Depression and fatigue were most predictive [40, 44]. Depression severity and worse work functioning were significantly associated with poorer SRCF in depressed patients [20]. For rheumatoid arthritis patients, sleep quality was significantly associated with SRCF [50]. Zhu, Hu, Xing, Guo, and Wu [51] reported that increased levels of HIV-related discrimination were associated with higher levels of SCRD even after controlling for demographics, mental health conditions, and social support.

#### 3.4.4. Severity of Chronic Conditions and Self-Reported Cognitive Function

Eleven studies (34%) examined associations between severity of chronic conditions and SRCF [21, 26, 32–35, 39, 41, 46, 47, 49]. Overall, multimorbidity and higher severity of disease were positively associated with greater self-reported cognitive problems. However, in one of these studies [39], those with type 2 diabetes mellitus (T2DM) and diagnosed cognitive impairment did not differ in the number of cognitive complaints when compared with those with T2DM who were cognitively healthy.

## 4. Discussion

Almost every evaluation of self-reported cognitive symptoms used a unique approach to assess self-reported cognitive function (28 of 32 studies, 88%). This heterogeneity of instrumentation can inhibit data sharing and generalizability of results across diverse populations. The use of common data elements for self-reported cognitive function in persons with nonneurologic chronic illness could contribute to accelerating intervention development and testing [13]. The reviewed studies used 28 measures to assess SRCF, with little overlap among them. A prior review and meta-analysis of studies (*n* = 53; 20,319 participants) examined the association between objective and subjective cognitive function in normatively aging adults without chronic illnesses and found that self-reported cognitive function accounted for less than 1% of the performance in objective measures [52]. However, that review included studies that used five specific measures for subjective memory and likely excluded a large number of other studies, because there are not any “gold-standard” assessments of self-reported cognitive function. We therefore expanded on that review in two ways: by focusing on self-reported cognitive function and a broader population, adults with at least one chronic condition. Two other meta-analyses of self-reported memory concerns and prediction of mild cognitive impairment and dementia (*n* = 49 studies) showed that conversion to dementia was 1.5 to 3 times higher in those who had self-reported cognitive complaints than in those who did not [53, 54]. Both reviews also noted the lack of established/standardized measures for self-reported cognitive function and the impact of depression on cognitive symptoms.

In the studies in this review, increased severity of chronic disease was associated with greater subjective cognitive impairment. Chronic diseases are themselves associated with a number of negative consequences such as lower quality of life, increased mortality, and loss of independence [55, 56]. A number of mechanisms dependent on the type of chronic illness may be responsible for this association. For example, in chronic obstructive pulmonary disease, low oxygen levels may directly affect the brain [57]. Or, more generally, physical illness can lead to fatigue and the subjective feeling of “not thinking well” [58]. It may also be that increased severity of disease has led to a decrease in leisure activities, exercise, sleep, or functional independence, which have protective effects on cognition [59]. Additionally, it has been shown that subjective cognitive dysfunction can impact daily self-management of chronic conditions like diabetes [10, 11] and quality of life [13, 59]. For these reasons, qualitative and quantitative research on subjective cognitive dysfunction in persons at risk for dementia are needed.

## 5. Conclusions

As this review demonstrates, many tools are used to measure self-reported cognitive symptoms, and clinicians should be aware that instrument selection will likely impact results. However, other reviews have found that self-reported cognitive complaints are a valid indicator of cognitive decline [60, 61]. It may be that deciding on a high-quality psychometric tool and using it consistently are important for clinical practice. Confounders for cognitive symptoms may also need to be assessed in clinical settings; anxiety, neuroticism, and dementia-related worry are variables related to increased subjective cognitive symptoms [60, 62].

Jessen et al. [63] provide a framework for investigating subjective cognitive symptoms in clinical settings and research. This framework includes suggested variables to examine (e.g., onset of subjective cognitive decline, believing one's cognitive performance is worse than of those of the same age) and criteria that increase the possibility of the existence of preclinical Alzheimer's disease in people with subjective cognitive decline. Molinuevo et al. [64] also suggest differentiating between “complaints” and “worries,” using a measure appropriate for the target population and including measures of stress, depression, and anxiety. Although the criteria in these two studies, from the Subjective Cognitive Decline Initiative Working Group, indicate that nonneurologic medical issues (e.g., chronic conditions) could underlie self-reported cognitive decline due to poor physical health, the studies do not suggest any recommendation other than to use care in interpreting the results of subjective cognitive complaints. Longitudinal assessment of subjective cognitive complaints may also be important, because changes in SRCF can indicate the functional benefit of prevention interventions.

### 5.1. Limitations

This review contributes a synthesis of measures and characteristics of self-reported cognitive symptoms in persons with nonneurologic chronic illnesses, but the heterogeneity of studies' effect sizes, outcomes, and measures did not permit data pooling, limiting cross study. The wide range of tests to measure perceived cognitive function likely contributed to variation in findings.

Cognitive impairment and chronic illness are both prevalent and detrimental. Given the present review's heterogeneity in assessment tools and evidence limitations, it is possible that self-management of chronic illness is influenced by the cooccurrence of cognitive symptoms. Future prospective longitudinal studies should examine the relationship of perceived cognitive symptoms and the self-management of chronic conditions, and assessments and interventions for improving cognitive function should be incorporated into care for older adults with chronic conditions.

## Figures and Tables

**Table 1:** Summary of included studies.

Author(s), year	Study design	Sample & setting	Assessment schedule	SRCF measure	Other assessment types	SRCF results	Association between SRCF and (1) NP tests (2) other PRO
Alonso-Prieto et al. [20]	Longitudinal	Depression *N* = 36 outpatient psychiatric clinic/Canada	Baseline and 8 weeks	British Columbia Cognitive Complaints Inventory (BC-CCI)	PRO	SRCF improved after treatment with desvenlafaxine (Cohen's d 1.24)	1. Not reported
							2. Significant association with work functioning and depression

Avants et al. [21]	Cross-sectional	HIV- v. HIV+*N* = 120 outpatient methadone maintenance clinic/United States	Baseline	Neuropsychological impairment scale	PRO	HIV +positive more cognitive impairment, cognitive symptoms, and intensity of symptoms (*F* = 0.6, *p* < .002; *F* = 10.7, *p* < .001; *F* = 3.8, *p* < .053)	1. Not reported
							2. SRCF significantly related to affective distress (r .880, *p* < .001)

Baker, Gibson, et al. [22]	Cross-sectional	Chronic pain	Baseline	Cognitive failures questionnaire, Everyday Memory Questionnaire behavior rating BRIEF-A (working memory subscale)	NP PRO	Group means for the SRCF measures indicated higher levels of reported problems (WM: 69.8(12.8) out of 100; CFQ: 51.18(21.56) out of 100; EMQ: 21.56(14.34) out of 52	1. SRCF was significantly correlated with NP tests (*p* < 0.5)
							2. Depression and catastrophizing did not moderate the association between subjective and objective performance
		*N* = 41					
		Community dwelling adults/Australia					

Baker, Georgiou-Karistianis, et al. [23]	RCT	Chronic pain	Baseline and 8 weeks	Cognitive failures questionnaire, Everyday Memory Questionnaire Behavior Rating Inventory of Executive Function	NP PRO	Intervention group improved SRCF v. control (ES .43, *p* = .017)	1. Not reported
		*N* = 39					2. Depression, anxiety, and pain interference, not significant
		Community dwelling adults/Australia					

Brück et al. [24]	Longitudinal	PTSD after ICU discharge	Baseline, 3, 6, and 12 months	Cognitive failures questionnaire	NP PRO	Prevalence of cognitive dysfunction 34% at 3 months, 51% at 6 months, and 45% at 12 months	1. Not significant
		*N* = 58					2. Not significant
		University hospital/Sweden					

Brunette et al. [25]	Longitudinal	Chronic obstructive pulmonary disease	Baseline and at 3 weeks	Cognitive difficulties scale (CDS)	NP PRO	No significant difference in SRCF between those with and without COPD	1. Cognitive difficulties were associated with worse performance (*p* .037)
		*N* = 59					2. Not significant
		Community based/United States					

Brunmeier et al. [26]	Cross-sectional	Congenital heart disease	Baseline	Functional assessment of cancer therapy (FACT) brain scale	PRO	34% met criteria based on SRCF for formal neuro cognitive evaluation	1. Not reported
		*N* = 337					2. Not reported
		Outpatient congenital heart program/United States					

Cockshell & Mathias [27]	Cross-sectional	Chronic fatigue	Baseline	Cognitive failures questionnaire	NP PRO	90% of those with chronic fatigue reported cognitive problems v. 12% without	1. Not significant
		*N* = 50					2. Depression was significantly positively related to SRCF (*p* < 0.01)
		Outpatient clinics/Australia					

Duijndam et al. [28]	Longitudinal	Cardiovascular disease	Baseline, 1 month, 12 months	Health complaints scale	PRO	Those with more perceived cognitive problems were younger and had more frequent percutaneous coronary intervention	1. Not reported
		*N* = 385					2. Poorer perceived cognition over time was related to poor quality of life (*p* < 0.01)
		Hospital-based/The Netherlands					

Fazeli et al. [29]	Cross-sectional	HIV	Baseline	Patient's assessment of own functioning inventory	NP PRO	Median SRCF score was 2 (0–9)	1. Significant association (*p* < 0.01)
		*N* = 100					2. Not reported
		Community dwelling/United States					

Frol et al. [30]	Cross-sectional	Asthma and rheumatoid arthritis	Baseline	Global measure of impairment (GMI; patient-rated)	NP PRO	65% taking corticosteroids had subjective cognitive problems v. 29% not taking corticosteroids	1. Not significant
		*N* = 31					2. Not significant
		Outpatient clinics/United States					

Gallo et al. [31]	Longitudinal	Cardiovascular disease	Baseline, 3.5–7.5 months	Cognitive difficulties scale (CDS)	PRO	Emotional distress and SRCF were significantly positively correlated (<.01)	1. Not reported
		*N* = 76					2. SRCF predicted emotional symptoms (*p* < 0.01)
		Outpatient cardiology clinics/United States					

Haley et al. [32]	Longitudinal	Cardiovascular disease	Baseline and 1 year	Cognitive difficulties scale (CDS)	PRO imaging	Higher baseline cognitive complaints were significantly related to lower cognitive ability at 12 months	1. Not reported
		*N* = 83					2. Cognitive complaints were significantly positively related to severity of microvascular disease (*p* = .028)
		Outpatient cardiology clinics and cardiac rehab/United States					

Henry et al. [33]	Longitudinal	End-stage kidney disease	Baseline, daily monitoring for 1 week	Cognitive function subscale of the kidney disease quality of life-short form	NP PRO	Ratings of cognitive impairment were greater on dialysis days when compared to nondialysis days (beta = 0.097, *p* = .005)	1. Greater diary-rated cognitive impairment was significantly related to lower working memory (beta = -0.07, *p* = .022), visual recall scores (beta = -0.05, *p* = .004), and longer dot tracing times (beta = 0.002, *p* = .005)
		*N* = 26					2. Not reported
		Dialysis clinic/United States					

Jackson & Cooper [34]	Cross-sectional	Diabetes, cardiovascular disease, arthritis, chronic obstructive pulmonary disease, obesity	Baseline	Investigator developed item “during the past 12 months, have you experienced confusion or memory loss that is happening more often or is getting worse?”	PRO	11.5% of the sample had experienced subjective cognitive decline in the preceding 12 months	1. Not reported
		*N* = 4,129					2. Those with subjective cognitive decline were significantly more likely to have depression (54.3%, *p* < 0.0001), be dissatisfied with life (24.7%, *p* < 0.0001), experience mental distress (37.6%, *p* < 0.0001), and feel they have inadequate social/emotional support (20%, *p* < 0.0001)
		Telephone survey/United States					

Jacob et al. [35]	Cross-sectional	Multiple chronic conditions	Baseline	Investigator developed item: “In the past month, have you had any problems with concentrating on what you were doing?” and “have you noticed any problems with forgetting things in the past month?”	PRO	The prevalence (95% CI) of subjective concentration complaints and subjective memory complaints was 22.0% (20.9–23.2%) and 29.9% (28.7–31.1%), respectively	1. Not reported
		*N* = 7,399					2. Depression and anxiety were significantly positively related to cognitive complaints (*p* < .001)
		Community based/United Kingdom					

Kiessling & Henriksson [36]	Cross-sectional	Coronary artery disease	Baseline	Cardiac health profile questionnaire (CHP)	PRO	No significant differences in assessed total SRCF scores between patients with or without a prior myocardial infarction (*p* = .78)	1. Not reported
		*N* = 253					2. SRCF was significantly correlated with quality of life (*p* < .001)
		In- and outpatient medicine departments/Sweden					

Kiessling & Henriksson [37]	Longitudinal	Coronary artery disease	Baseline, 1 year, 2 years	Cardiac health profile questionnaire (CHP)	PRO	Reduced perceived cognitive function [OR 1.59 (95% CI: 1.12–2.25); *p* = 0.0087] predicted sick leave or early retirement due to CAD	1. Not reported
		*N* = 169					2. Lower perceived cognitive function was associated with lower quality of life (*p* = .002)
		In- and outpatient medicine departments/Sweden					

Knoop et al. [38]	RCT	Chronic fatigue syndrome	Baseline, 8 months, 14 months	Checklist individual strength-concentration sickness impact profile-alertness behavior	NP PRO	Self-reported cognitive impairment decreased significantly more after CBT than in the control group	1. Not reported
		*N* = 233					2. Not reported
		Outpatient clinics/The Netherlands					

Matsuzawa et al. [39]	Cross-sectional	Type 2 diabetes	Baseline	Self-reported questionnaire for subjective complaints of memory and daily functioning: 3 items (yes/no) derived from the Cambridge examination for mental disorders of the elderly	NP PRO	Self-perception of memory dysfunction was not different between diabetic and nondiabetic participants (60.0% v. 60.0%)	1. Not reported
		*N* = 261					2. Memory dysfunction noticeable by others (*P* = 0.018) and impaired activity in taking medication (*P* = 0.001) predicted dementia
		Outpatient clinic/Japan					

McCracken & Iverson [40]	Cross-sectional	Chronic pain	Baseline	Sickness impact profile (SIP): alertness behavior subscale	PRO	54% reported at least one cognitive complaint. Most common subjective cognitive complaints: Forgetfulness (23.4%); minor accidents (23.1%); difficulty finishing tasks (20.5)	1. Not reported
		*N* = 275					2. Pain-related anxiety and depression were moderately associated with total cognitive complaints (*p* < .01)
		Outpatient clinic/Canada					

Morgan et al. [41]	Cross-sectional	Lower limb loss (vascular etiology)	Baseline	Quality of life in neurological disorders applied cognition–general concerns v1.0 short form	PRO	Subjective complaints were higher in those with limb loss v. controls	1. Not reported
		*N* = 484					2. Worse quality of life significantly associated with more cognitive complaints (*p* < .001)
		Community dwelling/United States					

Nguyen et al. [42]	Cross-sectional	Hypertension	Baseline	Subset of the memory functioning questionnaire (MFQ): 1-item on overall problems with memory	NP PRO	No significant difference in SRCF in those with hypertension v. those without hypertension	1. Those with memory complaints and hypertension had greater difficulty on NP tests than those without hypertension (*p* = .0003)
		*N* = 105					2. Not significant
		Community dwelling adults/United States					

Ott et al. [43]	Longitudinal	Depression	Baseline, 9 weeks, 14 weeks	Massachusetts general hospital cognitive and physical functioning questionnaire (CPFQ)	NP PRO	Those treated with erythropoietin had reduced cognitive complaints v. those not treated with erythropoietin	1. Not significant
		*N* = 79					2. Improvement in SRCF was not significantly associated with quality of life of occupational functioning
		Setting not described/Denmark					

Roth et al. [44]	Cross-sectional	Chronic pain	Baseline	Brief symptom inventory	PRO	62% reported moderate to severe problems with cognitive function	1. Not reported
		*N* = 222					2. Associations with negative affect, negative self, catastrophizing, neck pain, and fatigue were significant (*p* < 0.5)
		Outpatient pain management program/United States					

Sharma et al. [45]	Cross-sectional	HIV	Baseline	Self-reported cognitive complaints	PRO	12.5% reported subjective cognitive problems	1. Not reported
		*N* = 2,062					2. Subjective cognitive complaints were over twice as likely to report falls than those reporting no cognitive difficulties (AOR 2.19, 95% CI: 1.56–3.08)
		Community dwelling/United States					

Steinbusch et al. [46]	Longitudinal	Cardiovascular disease/cardiac arrest	Baseline, 2 weeks, 3 months, 1 year	Cognitive failures questionnaire	NP	Two weeks after cardiac arrest, SRCF was impaired in 11%, 12% at 3 months, and 14% at 1 year	1. Not reported
		*N* = 141					2. Not reported
		Inpatient coronary care units/The Netherlands					

Touradji et al. [47]	Cross-sectional	Lyme disease	Baseline	Questionnaire of neurocognitive complaints	NP	92% reported problems with cognitive function	1. Not significant
		*N* = 124					2. Not reported
		Outpatient clinic/United States					

Vance et al. [48]	Cross-sectional	HIV	Baseline	2003 AIDS Alabama needs assessment 4 items' assessing cognitive complaints	PRO	Mean cognitive complaints score was 17.63(5.57)–range 4–24 with higher scores indicating better SRCF	1. Not reported
		*N* = 427					2. Self-perceived health status and stress predicted SRCF (*p* < .05)
		Community AIDS services organization/United States					

Wingbermühle et al. [49]	Cross-sectional	Noonan syndrome	Baseline	Symptom checklist-90-revised (SC-90R)	NP PRO	Those with Noonan's reported more cognitive problems than control	1. There was significant difference in speed information processing (*F*_1,82_ = 5.15, *p* = .026, *η*_p_^2^ = 0.059) and delayed recall (*F*_1,82_ = 4.80, *p* = .031, *η*_p_^2^ = .055)
		*N* = 42					2. There was a significant difference in quality of life between groups (case group mean = 18.4, SD = 7.4; control group mean: 15.0, SD = 4.6; *t* (66.9) = 2.52, *p* = .014, d = 0.55)
		Medical center-genetics department/The Netherlands					

Yoon et al. [50]	Cross-sectional	Rheumatoid arthritis	Baseline	Perceived deficits questionnaire	NP PRO	Mean score on the PDQ was 11.8(5.1)	1. There was no significant relationship between total cognitive function score and SRCF score
		*N* = 40					2. Depression and sleep quality (*β* = 0.37, *p* = .025; *β* = 0.17, *p* = .034) were significantly associated with SRCF
		Outpatient rheumatology clinic/Korea					
Zhu et al. [51]	Cross-sectional	HIV	Baseline	AIDS health assessment questionnaire	PRO	47.22% reported at least one cognitive impairment in the last month	1. Not reported
		*N* = 324					2. Higher levels of perceived discrimination (*β* = −121, *p* = .036) were significantly associated with lower levels of SRCF
		Community clinic/China					

## Data Availability

The data are available in the submitted supplementary files.

## Supplementary Materials

Supplementary Materials Supplementary Materials. Supplementary Material A. Methods. Supplementary Material B. Search Strategy. Supplementary Material C. Data Extraction. Supplementary Material D. Quality Appraisals. Supplementary Material E. Study Demographics. Supplementary Material F. Table of Subjective Cognitive Function Measures.
