# Supplementary material for: Self-Reported Cognitive Function in Persons with Nonneurological Chronic Diseases: A Systematic Review
Source: J Aging Res. 2022 Mar 31;2022:5803337. doi: 10.1155/2022/5803337 (PMC8989496; doi:10.1155/2022/5803337)
Supplement: Supplementary Materials — Supplementary Material A. Methods. Supplementary Material B. Search Strategy. Supplementary Material C. Data Extraction. Supplementary Material D. Quality Appraisals. Supplementary Material E. Study Demographics. Supplementary Material F. Table of Subjective Cognitive Function Measures. [file 5803337.f1.doc]

**Supplementary Material** A. Methods

| **Review question:** | - What are the extent, quality and findings from available evidence regarding subjective cognitive function, objective, neuropsychological tests, and other patient reported outcomes in participants with non-neurologic chronic illnesses? |
| --- | --- |
| **Population:** | - Participants with non-neurologic chronic illnesses such as diabetes, cardiovascular disease and arthritis. |
| **Exposure:** | - N/A |
| **Comparators:** | - N/A |
| **Outcomes:** | - Must report primary study data or outcome data related to subjective cognitive function and neuropsychological or other patient reported outcomes. |
| **Inclusion criteria:** | - Primary study or secondary analysis/meta-analysis of primary study data [data-driven] - Peer-reviewed - English-language only - Published after 1990 - Population of interest includes individuals with non-neurologic chronic disease. |
| **Exclusion criteria:** | - Non data-driven study - Not peer-reviewed - Not written in English - Published before 1990 - Study does not focus on either 1) subjective cognitive function, or 2) lacks assessment with neuropsychological tests or other patient reported outcomes. - Study includes only participants with Alzheimer’s disease, stroke, fibromyalgia, traumatic brain injury or other neurologic conditions. |
| **Study selection:** | - Overall, 585 studies were identified through database searching and 281 through other sources. - After duplicates were removed, 304 studies remained. - 124 were excluded by title and abstracting screening. - 172 studies were assessed for eligibility via full-text screening, and 140 of these did not meet inclusion criteria, resulting in a total of 32 studies for analysis. |
| **Risk of bias assessment:** | - The quality of included studies was assessed with the Critical Appraisals Skills Programme (CASP, 2020), which includes eight appraisal tools to address common research study designs. For this review, randomized controlled trials (RCTs) were evaluated using the CASP RCT checklist, and other intervention studies were assessed using the CASP cohort study checklist for quasi-experimental studies. - Study quality was independently assessed by two reviewers using this methodological quality appraisal checklist. |

**Supplementary Material B. Search Strategy**

**Table 1. Medline** search strategy

| 1 | Chronic illness [Title/Abstract] OR chronic disease [Title/Abstract] OR non-dementia [Title/Abstract] |
| --- | --- |
| 2 | Subjective cognitive*[Title/Abstract] OR self-reported*[Title/Abstract] OR cognitive dysfunction [Title/Abstract] OR cognition*[Title/Abstract] OR subjective cognitive dysfunction [Title/Abstract] OR patient reported outcomes [Title/Abstract] |
| 3 | Neuropsychological tests [mesh] OR objective test* OR trial[mesh] |
| Filters | Publication date after 1989; Humans; English |

**Table 2. CINAHL search strategy**

| 1 | Chronic illness OR chronic disease OR non-dementia |
| --- | --- |
| 2 | Subjective cognitive* OR self-reported cognitive* OR cognitive dysfunction OR cognition* OR subjective cognitive dysfunction OR patient reported outcomes |
| **3** | Neuropsychological tests OR objective test* OR trial |
| Filters | Peer-reviewed, humans, English, after 1989 |

EMBASE, PubMed, and PsycINFO were searched using similar strategies

**Supplementary Material** C. Data Extraction

| 1 | Title |
| --- | --- |
| 2 | First author |
| 3 | Funding source |
| 4 | Possible conflicts of interest listed? (yes/no, if yes please list) |
| 5 | Addresses recruitment strategy? (yes/no) |
| 6 | Addresses retention strategy? (yes/no) |
| 7 | Addresses participant views on research? (yes/no) |
| 8 | Is the study guided by a theory or conceptual framework? (yes/no) |
| ***Demographics*** | |
| 9 | Does study focus on a specific population or subgroup? (yes/no, if yes please list) |
| 10 | Does study focus on participants living in a specific setting? – community, assisted living facility, skilled nursing facility, acute care |
| 11 | Inclusion criteria |
| 12 | Exclusion criteria |
| 13 | Total number of participants |
| 14 | Average age |
| 15 | Male (n, %) |
| 16 | Female (n, %) |
| 17 | White (n, %) |
| 18 | Racial/ethnic group membership |
| 20 | Aim of study |
| 21 | Design of study |
| 22 | Did the study use any questionnaires and/or tools? If so, note name here. |
| ***Subjective cognitive function*** | |
| 24 | Tool used to measure subjective cognitive function |
| 25 | Assessment schedule |
| 26 | Other assessment types (patient reported outcomes and/or neuropsychological tests) |
| 27 | Subjective cognitive function results |
| 28 | Associations between subjective cognitive function and (1) other patient reported outcomes (2) neuropsychological tests |
| 33 | Study findings |
| 34 | Study aim |
| 43 | Other key findings |
|  | Applicability |
| 44 | Have important populations been excluded from the study? |
| 45 | Do study findings directly address the review question? |
| ***Conclusion*** | |
| 46 | Key conclusion |
| 47 | Correspondence required for further study information? |
| 48 | Contact |
| 49 | Correspondence received |
| 50 | Notes |
| ***Other*** | |
| 51 | Study should be excluded as it does not meet eligibility criteria – put in check list of ineligibility |

**Supplementary Material D. Quality Appraisals**

Quality Appraisal of Cohort Studies

| Author,  Year | Clear aim | Acceptable recruitment | Exposure bias | Outcome bias | Identified factors | Adjusted for factors | Follow-up complete | Follow-up length | Results precise | Trust results | Applied locally | Fits other evidence |
| --- | --- | --- | --- | --- | --- | --- | --- | --- | --- | --- | --- | --- |
| Alonso-Prieto,  2019 |  |  |  |  |  |  |  |  |  |  |  |  |
| Avants, 1997 |  |  |  |  |  |  |  |  |  |  |  |  |
| Baker, Gibson  2018 |  |  |  |  |  |  |  |  |  |  |  |  |
| Brück, 2019 |  |  |  |  |  |  |  |  |  |  |  |  |
| Brunette,  2018 |  |  |  |  |  |  |  |  |  |  |  |  |
| Brunmeier,  2018 |  |  |  |  |  |  |  |  |  |  |  |  |
| Cockshell, 2013 |  |  |  |  |  |  |  |  |  |  |  |  |
| Duijndam,  2017 |  |  |  |  |  |  |  |  |  |  |  |  |
| Fazeli 2017 |  |  |  |  |  |  |  |  |  |  |  |  |
| Frol,  2013 |  |  |  |  |  |  |  |  |  |  |  |  |
| Gallo,  2015 |  |  |  |  |  |  |  |  |  |  |  |  |
| Haley,  2009 |  |  |  |  |  |  |  |  |  |  |  |  |
| Henry,  2011 |  |  |  |  |  |  |  |  |  |  |  |  |
| Jackson,  2017 |  |  |  |  |  |  |  |  |  |  |  |  |
| Jacob,  2019 |  |  |  |  |  |  |  |  |  |  |  |  |
| Kiessling,  2005 |  |  |  |  |  |  |  |  |  |  |  |  |
| Kiessling,  2004 |  |  |  |  |  |  |  |  |  |  |  |  |
| Matsuzawa,  2012 |  |  |  |  |  |  |  |  |  |  |  |  |
| McCracken,  2001 |  |  |  |  |  |  |  |  |  |  |  |  |
| Morgan,  2016 |  |  |  |  |  |  |  |  |  |  |  |  |
| Nguyen,  2016 |  |  |  |  |  |  |  |  |  |  |  |  |
| Ott,  2016 |  |  |  |  |  |  |  |  |  |  |  |  |
| Roth,  2005 |  |  |  |  |  |  |  |  |  |  |  |  |
| Sharma,  2016 |  |  |  |  |  |  |  |  |  |  |  |  |
| Steinbusch,  2017 |  |  |  |  |  |  |  |  |  |  |  |  |
| Touradii,  2019 |  |  |  |  |  |  |  |  |  |  |  |  |
| Vance,  2009 |  |  |  |  |  |  |  |  |  |  |  |  |
| Wingbemühle,  2012 |  |  |  |  |  |  |  |  |  |  |  |  |
| Yoon,  2017 |  |  |  |  |  |  |  |  |  |  |  |  |
| Zhu,  2016 |  |  |  |  |  |  |  |  |  |  |  |  |

Quality Appraisal of Randomized Controlled Trials

| Author,  Year | Clear aim | Random assignment | Attrition reported | Blind | Similar groups | Similar treatment | Effect | Precision of effects | Results applied locally | All outcomes | Benefits worth costs |
| --- | --- | --- | --- | --- | --- | --- | --- | --- | --- | --- | --- |
| Baker, Georgiou-Karistianis,  2018 |  |  |  |  |  |  |  |  |  |  |  |
| Knopp,  2017 |  |  |  |  |  |  |  |  |  |  |  |

Key

|  | Yes |
| --- | --- |
|  | Cannot Tell |
|  | No |

| **Supplementary Material E. Study Demographics** |  |  |  |  |  |  |
| --- | --- | --- | --- | --- | --- | --- |
| **Study** | **Sample Size** | **Country** | **Setting** | **% Female** | **Average Age**  **(*years*)** | **Race/ ethnicity** |
| Alonso-Prieto (2019) | N = 36 | Canada | Outpatient clinic | 58% | 38.7 | Not reported |
| Avants (1997) | N = 120 | United States | Outpatient clinic | 44% | 36.3 | Non-Hispanic white: 52%  African American: 36%  Hispanic: 10%  Unspecified: 2% |
| Baker, Gibson et al. (2018) | N = 41 | Australia | Community based | 62% | 42.9 | Not reported |
| Baker, Georgiou-Karistianis et al. (2018) | N = 39 | Australia | Community based | 63% | 43.1 | Not reported |
| Brück (2019) | N = 54 | Sweden | University hospital | 24% | Median age: 54  (range 41-64) | Not reported |
| Brunette (2018) | N = 59 | United States | Community based | 40.7% | 70.3 | Not reported |
| Brunmeier (2018) | N = 337 | United States | Outpatient clinic | 50% | 30 | Not reported |
| Cockshell (2014) | N = 50 | Australia | Outpatient clinic | 21% | 42.1 | Not reported |
| Dujndam (2017) | N = 385 | The Netherlands | Hospital based | 66% | 64.6 | Not reported |
| Fazeli (2017) | N = 100 | United States | Community based | 22% | 58.2 | Non-Hispanic white: 82%  Unspecified: 18% |
| Frol (2013) | N = 31 | United States | Outpatient clinics | 94% | 47.1 | Non-Hispanic white: 61.2%  African American: 29%  Hispanic: 6.5%  American Indian: 3.2% |
| Gallo (2005) | N = 76 | United States | Outpatient clinics | 22% | 64.3 | Not reported |
| Haley (2009) | N = 47 | United States | Outpatient clinics | 40% | 70.6 | Non-Hispanic white: 79%  African American: 2%  Unspecified: 19% |
| Henry (2018) | N = 26 | United States | Dialysis Centers | 57% | 42.7 | Non-Hispanic white: 7.7%  African American: 3.8%  Hispanic: 65.4%  Native American: 7.7%  Unspecified: 3.8% |
| Jackson (2017) | N = 4,129 | United States | Not reported | 60% | 45-54: 14.4%  55-64: 11.2%  65+: 9.2% | Non-white: 10%  White: 88.4%  Unspecified: 1.6% |
| Jacob (2019) | N = 7,399 | United Kingdom | Not reported | 51% | 46.3 | British white: 85%  British non-white: 15% |
| Kiessling (2004) | N = 253 | Sweden | In- and outpatient  medicine departments | 22% | 60.1 | Not reported |
| Kiessling (2005) | N = 169 | Sweden | In- and outpatient  medicine departments | 20% | 56.3 | Not reported |
| Knoop (2007) | N = 233 | The Netherlands | Outpatient clinics | 78% | 36.8 | Not reported |
| Matsuzawa (2012) | N = 261 | Japan | Outpatient clinics | 50% | 72.2 | Not reported |
| McCracken (2001) | N = 275 | Canada | Outpatient clinic | 62% | 46.6 | White: 75.3%  African American: 20.7%  Asian: 1.8%  Unspecified: 0.4% |
| Morgan (2016) | N = 484 | United States | Community based | 28.7% | 59.8 | Non-Hispanic white: 78.3%  African American: 12.2%  Hispanic: 5.8%  Unspecified: 5.8% |
| Nguyen (2016) | N = 105 | United States | Community based | 49% | 78.5 | White: 97%  African American: <3%  Asian: <3%  Unspecified: <3% |
| Ott (2016) | N = 79 | Denmark | Community based | Not reported | 41.9 | Not reported |
| Roth (2005) | N =222 | United States | Pain management program | 61% | 39.8 | White: 89%  African American: 8%  Asian: 1%  Unspecified: 3% |
| Sharma (20160 | N = 2,062 | United States | Not reported | 100% | <39: 16.7%  40-49: 36.1%  50-59: 37.6%  60+: 9.6% | White: 15%  African American: 73%  Unspecified: 12% |
| Steinbusch (2017) | N = 141 | The Netherlands | In-patient | 16% | 60 | Not reported |
| Touradii (2019) | N = 124 | United States | Outpatient clinics | 44% | 47.9 | Not reported |
| Vance (2009) | N = 427 | United States | Community based | 38% | 39.9 | Non-Hispanic white: 32%  African American: 63%  Other: 4% |
| Wingbermühle (2012) | N = 42 | The Netherlands | Genetics Department/Medical  Center | 57% | 30.6 | Not reported |
| Yoon (2017) | N = 40 | Korea | Outpatient clinic | 85% | 63.1 | Asian: 100% |
| Zhu (2019) | N = 324 | China | Community based | 35% | 55.8 | Han Chinese: 77%  Other: 23% |

**Supplementary Material F. Table of Subjective Cognitive Function Measures**

| **Measure or Tool Used** | **Number of Studies Using the Measure or Tool** | **Is this measure or tool validated or investigator designed?** | **Cognitive Domains Assessed** | **Reliability as reported by included study** | **Number of items and Scoring** | **Item Example** |
| --- | --- | --- | --- | --- | --- | --- |
| British Columbia Cognitive Complaints Inventory (BC-CCI) | 1 [20] | Validated | Concentration, Memory, Communication, Problem solving, | Not reported | 6 items  0 = not at all to 3 = very much; Higher scores indicate greater severity of cognitive problems | Not reported |
| Neuropsychological impairment scale | 1 [21] | Validated | Attention, Memory, Learning-Verbal | Sensitivity .91; specificity .76 | 95 items  0 = not at all to 4 = extremely; 3 summary scores (global impairment, total number of symptoms, symptom intensity); 7 clinical subscales (attention, memory, learning-verbal, frustration, academic); 3 test attitude scales (defensiveness, affective disturbance, internal consistency) | “I have trouble remembering important things”  “I like everyone I know”  “I feel easily annoyed and irritable.” |
| Cognitive Failure Questionnaire | 5 [22, 23, 24, 27, 46] | Validated | Attention, Concentration | Not reported | 25 items  0 = never to 4 = very often; score 0 – 100 with higher scores indicating more subjective cognitive problems | “Do you find you forget appointments?”  “Do you lose your temper and regret it?” |
| Everyday Memory Questionnaire | 2 [22, 23] | Validated | Memory, attention, working memory | Not reported | 13 items  5-point scale from “once or less in the last month to “once or more in a day” | Not reported |
| Behavior Rated Inventory of Executive Function | 1 [23] | Validated | Executive function | Not reported | 75 items  3-point scale: never, sometimes, often  Higher scores indicate worse subjective cognitive function | Not reported |
| Cognitive Difficulties Scale | 3 [25, 31, 32] | Validated | Attention, Language, Memory, Orientation, and Motor Functioning | Cronbach alpha = 0.96 | 39 items  5-point scale 0 = not at all to 4 = very often  Higher scores indicate more difficulty | “I have trouble recalling frequently used phone numbers”  “I have to do things very slowly to make sure I’m doing them right.” |
| Functional Assessment of Cancer Therapy (FACT) – Brain Scale | 1 [26] | Validated | Attention, Memory, Language, Executive function, Processing speed, Reading, Math | Not reported | 10 items  5-point scale  0 = not at all  4 = very much  Higher scores indicate fewer problems | “I am able to concentrate and/or sustain my attention”  “I am good at math” |
| Health Complaints Scale | 1 [28] | Validated | Concentration, Attention | Cronbach alpha = .67-.8 (concentration)  Cronbach alpha = .77 - .84 (attention) | 7 items  Concentration (item 1): 0 = not at all to 3 = almost everyday  Concentration (item 2): 1= not at all to 5 = extremely  Attention: 1= never or rarely true to 5 = very often or always true | “Trouble concentrating on things such as reading the newspaper or watching television”  “How well are you able to concentrate?”  “I do jobs or tasks automatically without being aware of what I am doing.” |
| Patient’s Assessment of Own Functioning Inventory | 1 [29] | Validated | Memory, Language, Use of hands, Sensory-perceptual, Higher level of cognitive and intellectual function, Work | Not reported | Number of items not reported  1 = almost never  6 = almost always  Higher scores indicate more symptoms | “How often do you forget something that has been told to you within the last day or two?” |
| Global Measure of Impairment (GMI – patient rated) | 1 [30] | Validated | Global | Not reported | Not reported | Not reported |
| Kidney Disease Quality of Life – Short form – cognitive function subscale | 1 [33] | Validated | Reaction time, Concentration, Confusion, Decision Making | Not reported | 4 items  6-point scale  1 = none of the time  6= all of the time  Higher scores indicate more cognitive difficulty | Not reported |
| “During the past 12 months, have you experienced confusion or memory loss that is happening more often or is getting worse?” | 1 [34] | Investigator designed | Confusion, Memory | Not reported | 1 item  Yes/No | N/A |
| “In the past month, have you had any problems with concentrating on what you were doing?” and “Have you noticed any problems with forgetting things in the past month?” | 1 [35] | Investigator designed | Concentration, Memory | Not reported | 2 items  Yes/No | N/A |
| Cardiac Health Profile Questionnaire | 2 [36, 37] | Validated | Concentration, Purposive Activity, Memory, Problem Solving | Cronbach’s alpha = .74 - .86 | 4 items  No scoring information | “How do you cope with tasks that require concentration and reflection?” “Do you easily forget things in the immediate past or where, for example, you have placed things?” |
| Checklist Individual Strength - Concentration | 1 [38] | Validated | Concentration, Memory | Not reported | 5 items  7-point scale | Not reported |
| Sickness Impact Profile – Alertness Behavior | 2 [38, 40] | Validated | Concentration, Alertness | Not reported | 10 items  Score ranges from 0 – 777  Higher scores indicate greater cognitive problems | “I sleep less at night and awaken frequently.” |
| Self-reported Questionnaire for Subjective Memory Complaints and Daily Functioning: 3 yes/no items derived from the Cambridge Examination for Mental Disorders of the Elderly | 1 [39] | Validated | Memory | Not reported | 3 items  Yes/No | “Do you have any complaints concerning your memory?” “Do you other people find you forgetful?”  “Do you often use notes to avoid forgetting things?” |
| Quality of Life in Neurological Disorders Applied Cognition – General Concerns v1.0 Short Form | 1 [41] | Validated | Memory, Attention, Decision making | Not reported | 8 items  Frequency of cognitive concerns over 7 days  Scores are centered on sample mean norms. Higher t-score indicates fewer cognitive concerns | “I had to read something several times to understand it.”  “I had trouble keeping track of what I was doing if I was interrupted.” |
| Memory Functioning Questionnaire – 1 item on overall problems with memory | 1 [42] | Validated | Global | Not reported | 1 item  1 to 7 scale  Higher scores indicate more cognitive problems | Not reported |
| Massachusetts General Hospital Cognitive and Physical Functioning Questionnaire | 1 [43] | Validated | Not reported | Not reported | Not reported | Not reported |
| Brief Symptom Inventory | 1 [44] | Validated | Memory, Concentration, Decision making | Cronbach’s alpha = .89 | 5 items  0 = not at all to 4 = extremely | Not reported |
| “Self-reported cognitive complaints” | 1 [45] | Investigator designed | Memory, Concentration, Confusion | Not reported | 2 items  Yes/No | Not reported |
| Questionnaire of neurocognitive complaints | 1 [47] | Investigator designed | Not reported | Not reported | 36 items  Cognitive symptoms are rated none, mild, moderate, or severe | Not reported |
| 2003 AIDS Alabama Needs Assessment: 4 items assessing cognitive complaints | 1 [48] | Validated | Problem solving, Memory | Cronbach’s alpha = .88 | 4 items  6-point scale  1 = all of the time to 6 = none of the time. | “During the past few weeks how much of the time did you experience the following: (1) difficulty solving problems, making plans, making decisions, learning new things, etc.?” |
| Symptom Checklist 90 – Revised | 1 [49] | Validated | Not reported | Not reported | Not reported | Not reported |
| AIDS Health Assessment Questionnaire | 1 [51] | Validated | Confusion, reaction time, concentration, reasoning, memory | Cronbach’s alpha = .91 | 5 items  0 = never  3 = severe  Higher scores indicate worse cognitive ability | Not reported |
| Perceived Deficits Questionnaire | 1 [50] | Validated | Attention, Concentration, Retrospective memory, Prospective memory, Planning | Not reported | 5 items  5-point scale  0 = never to 4 = almost always  Higher scores indicate greater perceived impairment over 4 weeks | “How often did you have trouble getting things organized?”  “How often did you have trouble concentrating on things like watching television or reading a book?” |
